# Morning Surge of Ventricular Arrhythmias in a New Arrhythmogenic Canine Model of Chronic Heart Failure Is Associated with Attenuation of Time-Of-Day Dependence of Heart Rate and Autonomic Adaptation, and Reduced Cardiac Chaos

**DOI:** 10.1371/journal.pone.0105379

**Published:** 2014-08-20

**Authors:** Yujie Zhu, Mohamed A. Hanafy, Cheryl R. Killingsworth, Gregory P. Walcott, Martin E. Young, Steven M. Pogwizd

**Affiliations:** Department of Medicine, University of Alabama at Birmingham, Birmingham, Alabama, United States of America; Brigham & Women’s Hospital - Harvard Medical School, United States of America

## Abstract

Patients with chronic heart failure (CHF) exhibit a morning surge in ventricular arrhythmias, but the underlying cause remains unknown. The aim of this study was to determine if heart rate dynamics, autonomic input (assessed by heart rate variability (HRV)) and nonlinear dynamics as well as their abnormal time-of-day-dependent oscillations in a newly developed arrhythmogenic canine heart failure model are associated with a morning surge in ventricular arrhythmias. CHF was induced in dogs by aortic insufficiency & aortic constriction, and assessed by echocardiography. Holter monitoring was performed to study time-of-day-dependent variation in ventricular arrhythmias (PVCs, VT), traditional HRV measures, and nonlinear dynamics (including detrended fluctuations analysis α1 and α2 (DFAα1 & DFAα2), correlation dimension (CD), and Shannon entropy (SE)) at baseline, as well as 240 days (240d) and 720 days (720d) following CHF induction. LV fractional shortening was decreased at both 240d and 720d. Both PVCs and VT increased with CHF duration and showed a morning rise (2.5-fold & 1.8-fold increase at 6 AM-noon vs midnight-6 AM) during CHF. The morning rise in HR at baseline was significantly attenuated by 52% with development of CHF (at both 240d & 720d). Morning rise in the ratio of low frequency to high frequency (LF/HF) HRV at baseline was markedly attenuated with CHF. DFAα1, DFAα2, CD and SE all decreased with CHF by 31, 17, 34 and 7%, respectively. Time-of-day-dependent variations in LF/HF, CD, DFA α1 and SE, observed at baseline, were lost during CHF. Thus in this new arrhythmogenic canine CHF model, attenuated morning HR rise, blunted autonomic oscillation, decreased cardiac chaos and complexity of heart rate, as well as aberrant time-of-day-dependent variations in many of these parameters were associated with a morning surge of ventricular arrhythmias.

## Introduction

Chronic heart failure (CHF), which affects over 5 million people in the US [1], is associated with increased incidence of sudden death primary from ventricular tachycardia (VT) degenerating to ventricular fibrillation [2]. A morning surge (between 6 AM and noon) in sudden deaths and ventricular arrhythmias have been demonstrated in patients with CHF [3]. The onset of other cardiovascular events such as heart attack, stroke and chest pain is also increased in the morning [4], [5], [6]. The underlying mechanisms are poorly understood, in part due to a lack of characterization of heart rate dynamics, autonomic oscillation and nonlinear dynamics in time-of-day-dependent adverse cardiac events in large animal CHF models. Moreover most studies to date have been done primarily in HF patients and have been limited and influenced by concurrent medication use.

Time-of-day-dependent variations in heart rate (HR) dynamics, autonomic nervous system and nonlinear dynamics are associated with the morning surge in cardiovascular events [3], [7]. Heart rate variability (HRV) can assess the regulation of arrhythmogenic substrate in CHF of the failing heart. Traditional linear HRV is analyzed in the time and frequency domain, and markers include SDNN (standard deviation of RR intervals), CV (coefficient of variation of RR intervals), and rMSSD (root mean square of successive differences), spectral power in different frequency range, etc. HRV has been shown to have important prognostic implications [8], [9], [10], [11], [12], [13]. Heart rate fluctuations have been recognized as complex dynamical behaviors originating from nonlinear processes [8], [10], [11], [12], [13], [14], [15], [16], [17]. Nonlinear dynamic approaches to HRV are used to determine if HRV has features typical of chaos (complexity & fractal-like behavior) [18]. Nonlinear measures study complex interactions of hemodynamic, electrophysiological, and humoral variables and their regulation by the autonomic and central nervous systems, and have been shown to have prognostic value in CHF [8], [9], [10], [11], [12], [13]. Cardiac chaos is decreased in human CHF [17] and greater reduction in parameters of cardiac chaos is associated with poorer prognosis in CHF patients [19]. Altered fractal properties, fractal-like scaling exponents and correlation properties of HRV, have been shown to precede the onset of lethal arrhythmias, changes that traditional (i.e. linear) HRV markers failed to detect [20], [21].

Despite knowledge of HRV parameters in patients with CHF which are associated with a morning surge in ventricular arrhythmias, the underlying mechanisms contributing to these important observations have remained elusive. We have recently developed a novel arrhythmogenic large animal model of CHF in the canine heart that exhibits decreased LV function and spontaneous ventricular arrhythmia that are initiated and maintained by a focal nonreentrant mechanism [22]. The purpose of the present study was to assess whether there is a morning surge in premature ventricular complexes (PVCs) and VT in our new irreversible arrhythmogenic canine CHF model, and to determine whether this morning surge is associated with alterations in heart rate dynamics, autonomic nervous system and nonlinear dynamics of HRV.

## Methods

### Animals

All experiments were carried out in strict accordance with the recommendations in the Guide for the Care and Use of Laboratory Animals of the National Institutes of Health (NIH Publication No. 85-23, revised 1996) [23]. The protocols were approved by the Institutional Animal Care and Use Committee of the University of Alabama at Birmingham. Mixed breed dogs of both sexes were housed individually in metal cages with a 12-hr light on (6∶00–18∶00)/12-hr light off cycle. Neither control dogs nor CHF dogs were on any medication chronically.

### Induction of chronic heart failure in dogs

Heart failure was induced by combined aortic insufficiency (AI) and abdominal aortic constriction (AC). For creation of aortic insufficiency, dogs were given Telazol (6 mg/kg, IV) and atropine (0.04 mg/kg, IM). Cefazolin (25 mg/kg IM) and 0.1–0.2 mg/kg Buprenorphine (IM) were subsequently admininistered. The dogs were intubated and ventilated with isoflurane/O_2_ mechanically during the surgery. Under continued fluoroscopic imaging, AI was induced by perforating the right and non-coronary aortic valve leaflets using a 5F catheter and a custom-shaped trochar. A 4F Fogarty balloon catheter was passed through the perforation over a guide wire. The balloon was inflated and pulled through the valve to enlarge the perforation. Based on our well-established CHF rabbit model [24], sufficient AI was achieved when the aortic pulse pressure increased by a minimum of 50%. This was confirmed by color flow echocardiography. Eleven dogs were serially studied long term up to 720 days while 15 dogs were used for other terminal studies or died prior to 720 days after AC.

Aortic constriction (AC) to cause pressure overload was created ∼6 weeks later, when M-mode echocardiographic measurements indicated that the LV end-diastolic diameter (LVEDD) had increased by 20% compared to baseline. The same anesthesia protocol was used during the surgery. After midline laparotomy, the aorta was isolated at the level of the left renal artery. A 2–0 silk ligature or a sterile polyethylene band was tightened to reduce the aortic diameter until the systolic blood pressure gradient was increased by ∼45–50 mm Hg from the pre-ligation measurement. In two dogs, a ligature was also placed around a branch of the renal artery. The aortic systolic pressure gradient was determined using a 4 F catheter passed through a vascular sheath in the femoral artery.

### Echocardiography

Prior to any surgical intervention and at approximately 240 days and 720 days following AC, dogs underwent M-mode, 2-D, and color flow echocardiography (sedated with 0.1 mL butorphenol i.v.) using a Philips SONOS 5500 ultrasound system. LV end-systolic (LVESD) and end-diastolic diameter (LVEDD) were measured and the LV fractional shortening (FS = [LVEDD–LVESD]/LVEDD×100]) was calculated.

### Holter monitoring

Prior to surgical intervention and at 240 days and 720 days post AC, digital Holter monitoring (GE SEER Light, GE Healthcare) was obtained from dogs in the conscious state. The chest and back were shaved and five ECG electrodes were applied to the chest secured by a specially-designed nylon jacket. 24-hour period Holter recording were made with the dogs’ movements being unrestricted. Holter data were analyzed to quantitate VT & PVCs and their time-of-day occurrence. VT episodes (3 or more consecutive beats), VT beats and PVCs were confirmed by an experienced cardiologist.

### HRV analysis

ECG data from Holter recordings were digitized with a sampling rate at 125 Hz. The RR intervals and annotation of heart-beats were exported from the GE MARS Holter system for further analyses using a specially-designed Matlab-based program, as well as Matlab-based Kubios program [25]. Ectopic beats were excluded from HRV analysis.

Power spectral density was assessed using parametric autoregressive (AR) modeling-based methods. Low frequency power (LF, 0.04–0.15 Hz) mainly reflects sympathetic nerve activity (SNS). High frequency power (HF, 0.15–0.40 Hz) primarily reflects parasympathetic (PNS) modulation of HR. While LF/HF (ratio of LF to HF) have been used as a direct reflection of the ratio of sympathetic to parasympathetic input, it appears to be more of an indicator of overall balance between SNS and PNS [25], [26], [27]. For example, LF/HF ratio is higher when SNS is predominant, whereas a small LF/HF ratio suggests predominantly PNS control.

Correlation dimension is a measure of dimensionality of the space occupied by a set of points that can be used to measure complexity of the RR interval time series [25], [28], [29]. Reconstruction of the attractor (vectors with a set of length embedded in m-dimensional space) was performed. Then the correlation integral was calculated with the number of points in the phase space that are closer than a certain threshold r, repeating the process for a certain range of thresholds. Correlation dimension was computed as the slope of the line fitting the log-log plot of the correlation integral as a function of threshold if the number of points was sufficiently large and r was small.

Detrended fluctuation analysis (DFA) quantifies fractal-like scaling exponents and correlation properties of RR intervals [8], [25], [30]. The RR interval time series was integrated, and detrended within each segment. The root mean square fluctuation of the integrated and detrended data was repeatedly measured over different segment lengths and plotted against different segment lengths on a log-log scale. The scaling exponent DFA α indicates the slope of this regression line. The short-term fluctuation DFA α1 was calculated within range 4≤n≤16 and long-term fluctuation DFA α2 was calculated within range 16≤n≤64.

Shannon entropy (SE) is a measure of signal complexity of the time series dynamics, and is the information entropy of line segment density distributions from the RR intervals [25], [31]. It is measured by the negative sum of the multiplication of the probabilities and base 2 logarithms of probabilities for given line lengths. The probability is the number of length lines divided by the total number of lines.

### Statistical analysis

Data are reported as means ± SEM. One-way analysis of variance (ANOVA) was used for measurements obtained from multiple groups. Comparisons between values on two individual groups were done using the two-group (unpaired) t test. Cosinor method for computing rhythm analysis was performed using the nonlinear regression function by SPSS. Rhythmicity was evaluated by fitting the nonlinear cosine function, and a HRV parameter was considered to have a significant time-of-day-dependent oscillation if it fit a cosine wave function for p<0.05, with a periodicity of approx. 24 hours. X^2^ goodness-of-fit testing was used to determine non-uniform distribution of timing in the 24-hour period.

## Results

### CHF dogs developed progressive cardiac enlargement & LV dysfunction

**Figure 1:**
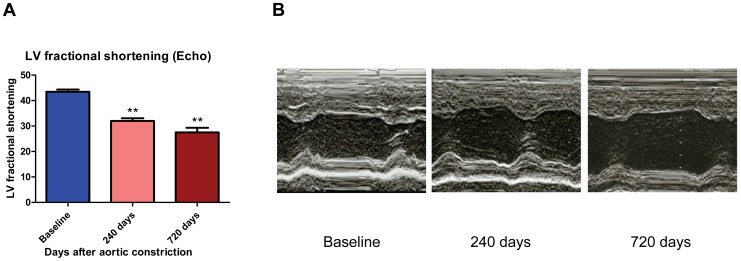
CHF dogs developed LV dysfunction. (A) Histogram of LV fractional shortening (FS) at baseline, 240 days and 720 days after AC (* *, p<0.01 vs baseline). (B) 2D echocardiography demonstrates increased LV end-diastolic dimension (LVEDD) and LV end-systolic dimension (LVESD) in CHF dogs.

Compared with baseline, CHF dogs developed progressive cardiac enlargement and decreased left ventricular (LV) systolic function following AI/AC, as assessed by echocardiography. LV end-diastolic dimension (LVEDD) progressively increased by 59 and 66% after 240 and 720 days post-AC, respectively. Likewise, LV end-systolic dimension (LVESD) increased by 91 and 114% after 240 and 720 days post-AC, respectively. LV fractional shortening progressively decreased from 43.5±0.9% at baseline to 32.0±1.1% at 240 days and 27.5±1.8% at 720 days after AC in CHF dogs (Figures 1 A&B).

### Number of VT episodes, VT beats and PVCs show morning rise in CHF dogs

**Figure 2:**
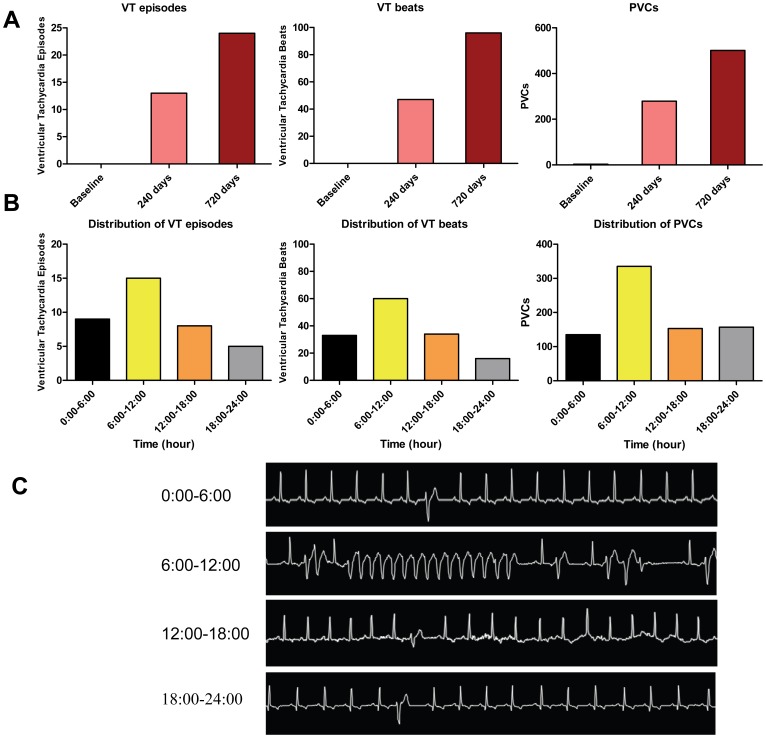
VT episodes, VT beats and PVCs show morning rise in CHF dogs. (A) There is progressive increase in ventricular arrhythmias (VT episodes, VT beats and PVCs) with the duration of CHF. (B) Both VT episodes and VT beats showed a morning rise (1.7-fold & 1.8-fold increase at 6 AM-noon vs midnight-6 AM), and PVCs showed a morning rise (2.5-fold increase at 6 AM-noon vs midnight-6 AM). (C) Representative spontaneously-occurring VT and PVCs in a CHF dog.

At baseline, there were no episodes of VT and only a total of 3 PVCs in 26 dogs. The incidence of VT episodes, VT beats and PVCs assessed by 24-hr Holter monitoring progressively increased at 240 and 720 days (Figure 2A). Moreover, ventricular arrhythmias exhibited a marked diurnal variation with a rise during the morning in CHF dogs. The number of VT episodes, VT beats and PVCs during 6∶00 AM to 12∶00 PM was elevated compared with the other time of day (Figure 2B). VT episodes, VT beats and PVCs occurred 1.7, 1.8 and 2.5 times more frequently during 6∶00 AM to 12∶00 PM than the preceding quartile (between 12∶00 AM and 6∶00 AM), and all showed a morning rise during 6∶00 AM to 12∶00 PM at 0.05 significance level by χ^2^ goodness-of-fit test. Figure 2C showed representative spontaneously-occurring VT and PVCs in CHF dogs.

### Morning (6 AM-noon) HR rise is attenuated in CHF

**Figure 3:**
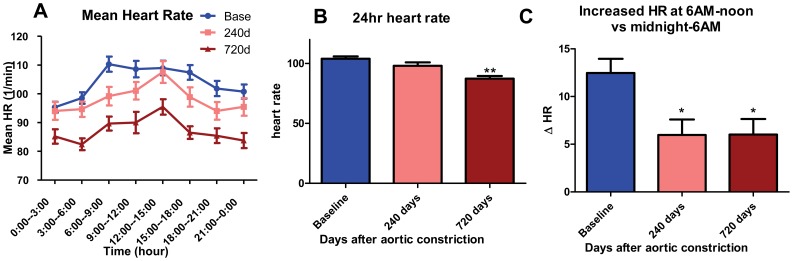
Heart rate at baseline and CHF. (A) Summarized data for mean heart rate at baseline, 240 days and 720 days after AC. Mean heart rate is the average of every 3 hours. (B) Histogram of average 24-hour HR at baseline, 240 days and 720 days after AC (**, p<0.01 vs baseline). (C) Histogram of ΔHR at baseline, 240 days and 720 days after AC (*, p<0.05 vs baseline). ΔHR represents the increased average heart rate at 6 AM-noon vs midnight-6 AM.

The average heart rates (HR) for every 3 hours intervals of the day at baseline (N = 26 dogs), and at 240 days (N = 18) and 720 days (N = 11) after AC, are shown in Figure 3A. At baseline, it appears that HR rises rapidly in the morning (with a 13% increase in HR from 6 AM-noon vs midnight-6 AM), which is not observed in the CHF groups at 240 and 720 days. With CHF, the morning HR rise was not as great (ΔHR for 6 AM-noon vs midnight-6 AM of 5.97±1.63 and 6.02±1.62 bpm for 240 & 720 days post-AC, respectively, vs 12.47±1.48 bpm at baseline, p<0.05 & 0.05, N = 18, 11, 26; Figure 3C). With development of CHF, mean HR (over 24 hrs) remained unchanged at 240 days but was decreased at 720 days vs baseline (87±2 vs 104±2 bpm, p<0.01, N = 11, 26), likely reflecting sinus node dysfunction that is well-described in CHF [32], [33].

### Morning ANS adaptation is attenuated with CHF

**Figure 4:**
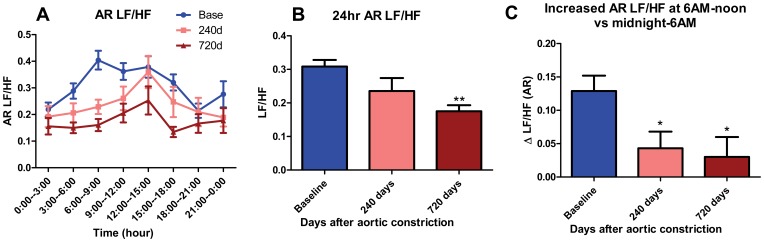
LF/HF at baseline and CHF. (A) Summarized data for mean LF/HF at baseline, 240 days and 720 days after AC. Mean LF/HF is average of every 3 hours. (B) Histogram of average 24-hour LF/HF at baseline, 240 days and 720 days after AC (**, p<0.01 vs baseline). (C) Histogram of Δ LF/HF at baseline, 240 days and 720 days after AC (*, p<0.05 vs baseline). Δ LF/HF represents the increased average LF/HF at 6 AM-noon vs midnight-6 AM.

From spectral analyses, the ratio of the low frequency to high frequency (LF/HF) components, a measure of autonomic balance between sympathetic and parasympathetic nervous systems, appears to rise rapidly during the morning at baseline (6 AM-noon vs midnight-6 AM; p<0.01 by ANOVA; N = 26, Figure 4A). There was attenuation of the morning rise in LF/HF (from 6 AM-noon vs midnight-6 AM) (Figure 4C) at 240 & 720 days in CHF dogs. With CHF there was a progressive decline in LF/HF such that by 720 days post-AC LF/HF had decreased by 43% (p<0.01 by ANOVA; N = 26, 11, Figure 4B).

### Correlation dimension progressively decreases with CHF with attenuation of morning rise

**Figure 5:**
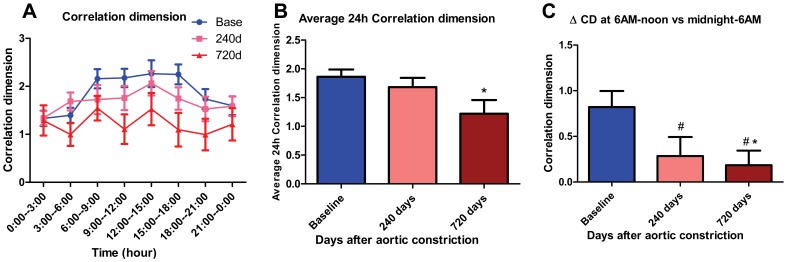
Correlation dimension (CD) decreases with CHF. (A) Summarized data for mean CD at baseline, 240 days and 720 days after AC. Mean CD is average of every 3 hours from 24-hour recordings. (B) Histogram of average 24-hour CD at baseline, 240 days and 720 days after AC (*, p<0.05 vs baseline). (C) Histogram of Δ CD at baseline, 240 days and 720 days after AC (*, p<0.05 vs baseline; # denotes that CD is arrhythmic at 240d and 720d by rhythm analysis, i.e. exhibits loss of time-of-day dependence). Δ CD represents the change in CD at 6 AM-noon vs midnight-6 AM, and was decreased with CHF.

CD is one of the nonlinear parameters reflecting cardiac chaos, a decrease of which can be detrimental [34]. Figure 5A shows that CHF provoked consistent progressive reductions in CD. Average 24-hour CD decreased with HF such that by 720 days, CD was reduced by 34% (1.22±0.24 at 720 days vs 1.86±0.13 at baseline, p<0.05, N = 11, 26, Figure 5B). At the time of enhanced arrhythmia occurrence (6 AM-noon), CD was reduced by 39% (1.33±0.19 at 720 days vs 2.18±0.17 at baseline, p<0.01, N = 11, 26), and there was attenuation of the morning rise in CD both at 240 and 720 days (Figure 5C). Moreover, cosinor analysis revealed that CD significantly oscillated with a periodicity of approx. 24 hours at baseline (p<0.01), which was lost at 240 & 720 days (p>0.05, p>0.05).

### Detrended fluctuations analysis (DFA) α1 and α2 shows progressively decreased fractal properties with CHF with attenuation of morning rise

**Figure 6:**
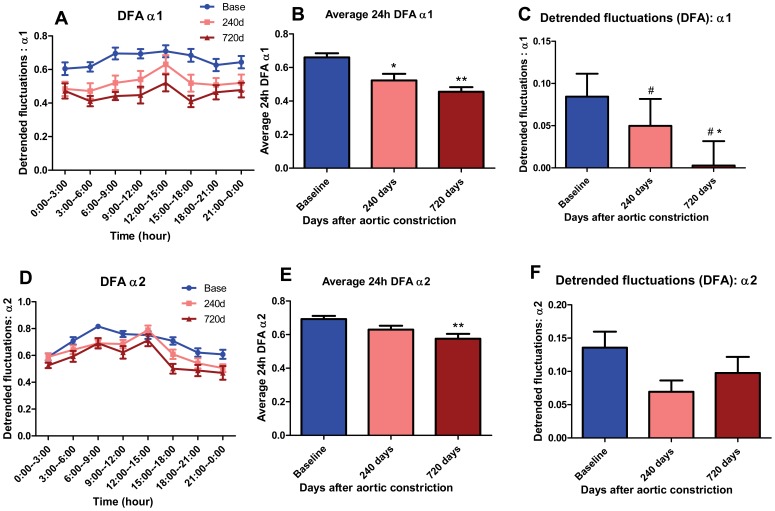
Detrended fluctuations analysis (DFA) α1 and α2. (A) Summarized data of average DFA α1 at baseline, 240 days and 720 days after AC. Mean DFA α1 was the average of every 3 hours. (B) Histogram of average 24-hour DFA α1 at baseline, 240 days and 720 days after AC (*, p<0.05 vs baseline; **, p<0.01 vs baseline). (C) Histogram of ΔDFA α1 at baseline, 240 days and 720 days after AC (*, p<0.05 vs baseline; # denote that DFA α1 is arrhythmic at 240d and 720d by rhythm analysis, i.e. exhibits loss of time-of-day dependence). ΔDFA α1 represents the increased average heart rate at 6 AM-noon vs midnight-6 AM, and was decreased with CHF. (D) Summarized data of average DFA α2 at baseline, 240 days and 720 days after AC. Mean DFA α2 is average of every 3 hours. (E) Histogram of average 24-hour DFA α2 at baseline, 240 days and 720 days after AC (* *, p<0.01 vs baseline). (F) Histogram of ΔDFA α2 at baseline, 240 days and 720 days after AC. ΔDFA α2 was decreased in CHF. ΔDFA α2 represents the change in DFA α2 at 6 AM-noon vs midnight-6 AM.

DFA α1 and α2 reflect the fractal properties of heart rate. Reduced DFA α1 was reported to be a very powerful measure as a predictor of all-cause mortality [8]. Figures 6A and 6D show that at baseline there was a morning rise in DFAα1 and DFAα2 (Figures 6A&D). With CHF the average 24-hour DFA α1 was significantly decreased at 240 and 720 days (0.523±0.039 and 0.456±0.027 at 240 & 720 days, p<0.05 & p<0.01, N = 18, 11) vs 0.660±0.025 (N = 26) at baseline (Figure 6B). Average 24-hour DFA α2 was significantly decreased only at 720 days vs baseline (0.575±0.029 vs 0.692±0.019 at baseline, p<0.01, N = 11, 26; Figure 6E). The potential protective effect of enhanced DFA α1 at the time of increased arrhythmia occurrence was greatly attenuated with CHF (Figure 6C). Rhythm analysis by cosinor method showed that DFAα1 is rhythmic at baseline (p<0.05), and loses time-of-day-dependent oscillation at 240d and 720d after AC in CHF dogs (p>0.05, p>0.05). In contrast there was no significant difference in the morning rise of DFAα2 between baseline and CHF (Figure 6F).

### Shannon entropy shows progressive decrease with CHF and lack of morning surge

**Figure 7:**
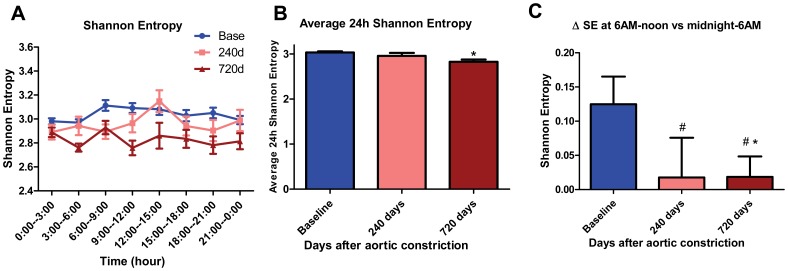
Shannon entropy (SE) decreases with CHF. (A) Summarized data for mean SE at baseline, 240 days and 720 days after AC. Mean SE is the average of every 3 hours. (B) Histogram of average 24-hour SE at baseline, 240 days and 720 days after AC (*, p<0.05 vs baseline). (C) Histogram of Δ SE at baseline, 240 days and 720 days after AC (*, p<0.05 vs baseline; # denotes that SE is arrhythmic at 240d and 720d by rhythm analysis, i.e. exhibits loss of time-of-day dependence). Δ SE, which represents the change in SE at 6 AM-noon vs midnight-6 AM, was decreased in CHF.

SE is another analytical approach for complexity of time series, and reduced SE has been reported in heart disease [35]. Figure 7A illustrates that at baseline there was a small morning rise in SE (Figure 7A). With CHF, average 24-hour SE decreased by 7% at 720 days post-AC (2.828±0.051 vs 3.037±0.022 at baseline; p<0.05; N = 11, 26; Figure 7B). However the morning rise in SE was practically abolished with CHF (Figure 7C). Cosine curve fitting in SE showed time-of-day-dependence at baseline (p<0.05), but loss of time-of-day-dependent variations at 240 and 720 days (p>0.05, p>0.05).

## Discussion

Morning is a time of increased workload, physical activity and heart rate and sympathetic activity [36], associated with increased incidence of adverse cardiac events. In this study we explored linear and nonlinear dynamics of HRV and their time-of-day-dependent variation in a novel irreversible large animal model of nonischemic CHF in dog. We show, in a large animal model of CHF: a morning surge in ventricular arrhythmias with CHF; a morning rise in HR at baseline that was attenuated in CHF; a morning rise in LF/HF (balance between sympathetic vs parasympathetic system) that was blunted in CHF; reduced chaos in CD, DFA α1 & α2 and SE in CHF; a loss of time-of-day dependence was found in LF/HF, CD, DFA α1 and SE in CHF; and a lack of enhanced chaos at the time of enhanced arrhythmia occurrence. Several unique features of this study include this being the first demonstration of time-of-day-dependent pattern of VT and PVCs in a large animal model of CHF, and this being the first study to characterize chaotic behavior of HRV and its changes with CHF in the canine heart and demonstrating findings similar to those shown in human CHF [30], [34], [35], [37], [38], [39], [40], [41].

### Normal time-of-day rhythm

At baseline, dogs show normal left ventricular (LV) systolic function and exhibit lack of cardiac arrhythmia. At baseline state, morning HR significantly increased during 6∶00 AM to 12∶00 PM compared to midnight-6∶00 AM. Dogs at baseline also had a significantly higher LF/HF rise in morning (vs 12∶00 AM–6∶00 AM) compared to CHF dogs. These findings are similar to reports in healthy human subjects [42]. The values of nonlinear dynamics such as CD, DFA α1 and SE are higher at baseline compared to CHF dogs. Cosinor analysis revealed that CD, DFA α1 and SE showed significant time-of-day-dependent oscillation at baseline with a periodicity of approximately 24 hours.

### HF model in absence of drug effects

Studies from HF patients have provided important insights into the alterations in autonomic influences that contribute to the arrhythmogenic substrate of the failing heart [8], [10], [11], [12], [14], but these studies are typically limited by concurrent medication use such as beta blockers, ACE inhibitors and Ca^2+^ channel blocker. Holter monitoring of HF patients with assessment of HRV to study autonomic imbalance as well as nonlinear dynamics will be influenced by these medication and their drug effects. It is important and essential to study electrophysiological and autonomic regulation in chronic heart failure in the absence of drug effects. This is feasible with studies of HRV (in absence of drug effects) by using a large animal model of CHF that is similar to human nonischemic CHF and which can be used for development of new therapies and for translating basic scientific discoveries from bench to bedside. While there are a number of large animal models of nonischemic HF, the canine rapid pacing HF model is one of the most commonly used. It has provided insights into the complex pathophysiology of HF. However, limitations of the canine pacing HF model include the reversibility of HF with cessation of pacing (making long-term studies of interventions on the failing heart difficult), remodeling different from human CHF, and the lack of hypertrophy [43]. We developed a novel canine model of nonischemic CHF that is both irreversible and arrhythmogenic by induction of combined aortic insufficiency (AI) and abdominal aortic constriction (AC). The hemodynamic burden of combined volume and pressure overload in this canine heart leads to irreversible contractile dysfunction and arrhythmogenesis. We have found that our CHF dogs exhibit a 63% increase in HW/BW [44] and a 66% increase in LVEDD. Moreover this canine model of CHF demonstrates a morning surge in ventricular arrhythmias as well as reduced chaos that will enable us to further define underlying mechanisms in the absence of drug effects so commonly an issue in CHF patients.

### Morning surge in adverse cardiovascular event

Prior studies have highlighted the excess risk of adverse cardiovascular events during morning. For example, sudden cardiac death exhibits an increased frequency in the morning [45]. The majority of VT episodes in patients with dilated cardiomyopathy occurred during morning [46], with an increase in the onset of sustained VT at that time [47]. PVCs also increase in the morning [48], [49]. In addition to the morning surges in sudden death, VT and PVCs, numerous studies have shown similar morning rise in other cardiovascular events such as chest pain [6], myocardial infarctions [4], [6], and stroke [5]. This time-of-day-dependent pattern of excess cardiovascular risk during the morning associated with awakening is an appealing target for therapeutics. However, the lack of a suitable animal model of this phenomenon has hindered our understanding of the mechanisms contributing to the morning rise in these adverse events. Demonstration of time-of-day-dependent oscillations of VT and PVCs in a large animal model of CHF in the current study provides a unique opportunity to explore underlying mechanisms in the future.

### Factors that may be associated with morning surge and time-of-day-dependent variations

Morning rise in heart rate was attenuated in CHF dogs, similar to that in patients with stable coronary artery disease [41], [50] and CHF [39], and may be related to altered contribution of autonomic system in the morning or sinus node dysfunction in CHF [32], [33]. We found that the morning rise in LF/HF was attenuated in CHF, similar to that shown in patients with coronary artery disease [38] and in patients with morning peak of tachyarrhythmic events [51], [52]. Reduced LF power was associated with increased risk of ventricular arrhythmias and cardiac death in patients with CHF in several studies [9], [53], [54], [55], [56], [57], [58]. We also found reduced chaos in CHF, a lack of enhanced chaos at the time of enhanced arrhythmia occurrence, and a loss of time-of-day-dependent variations for LF/HF, CD, DFA α1 and SE in CHF. Non-linear dynamics and chaotic indices in HRV were significantly reduced in patients with a propensity for adverse arrhythmic events which exhibit morning surge [20], [59], [60], [61], [62], [63]. Reduced chaos was associated with increased arrhythmias [63], [64], [65]. Thus the changes in linear and nonlinear dynamics in our CHF dogs are similar to that in CHF patients, thereby validating this new arrhythmogenic canine preparation as a valuable model in which to further study molecular, cellular, physiologic and neurohumoral mechanisms in ways not possible in humans.

There are numerous factors that may contribute to this time-of-day elevated incidence in ventricular arrhythmias in dogs as well as in humans. Catecholamines, angiotensin, renin, aldosterone and growth hormone [6] have higher plasma concentrations in morning during maximal sympathetic activity in humans [6]. Protective regulators such as bradykinin, magnesium, potassium, vitamin E and C and melatonin are all low in the morning [6], and may be associated with greater vascular resistance, blood pressure, contractility, coronary artery tone, stroke volume and cardiac output [6]. Additional factors such as acetylcholine, histamine, serotonin, and myocardial oxygen demand [6], [41], [66] exhibit circadian variation and may contribute to increased risk of adverse cardiovascular events in the morning. All these corresponding biochemical changes in the morning in CHF dogs are reflected by attenuated morning HR rise, blunted autonomic oscillation, decreased cardiac chaos, a loss of time-of-day-dependent rhythm, and a lack of enhanced chaos at the time of enhanced arrhythmia occurrence. Diurnal variation of HRV was reported in healthy human subjects [37], [67], [68]. We found loss of diurnal variation of HRV in CHF dogs as has been described in human HF [37]. Likewise we found a consistent and progressive decrease in correlation dimension, DFA scaling factor α and SE in CHF dogs as has been shown in human HF [30], [34], [35], [40].

### Heart failure and autonomic system

In the current study, the morning rise in LF/HF was attenuated with development of CHF at 240 days and 720 days. Reduction in LF/HF was found in CHF dogs. These may reflect sinus node dysfunction, decreased responsiveness of the sinus node to autonomic input [69] or a CHF-induced reduction in baroreceptor sensitivity in the setting of increased sympathetic tone that has been consistently found in HF [69], [70]. Several studies demonstrate that LF power is greatly attenuated [71] and the LF variability of sympathetic nerve activity is absent in patients with heart failure [72], [73]. Reduced LF is a powerful predictor of sudden death in CHF patients [9]. In CHF dogs, there was attenuation of the morning rise in LF/HF. On the other hand, ventricular arrhythmias were preceded by elevated sympathetic activation [74], [75]. Increased PVCs and VT may correspond to higher sympathetic nerve activation in morning hours [74].

### Reduced chaos in heart failure

Several studies have demonstrated reduced complexity and fractal properties in CHF patients. Poon et al found a decrease in complexity of the heartbeat nonlinear dynamics in CHF patients [17]. Ivanov demonstrated that patients with severe heart failure have a reduced HRV complex. CHF patients show a loss of multifractality [16]. DFA α1 remained as a useful prognostic index in multivariate analysis and was a powerful predictor of mortality among class II CHF patients [11]. Patients with cardiovascular disease showed reduced scaling exponents, suggesting a loss of fractal-like HR dynamics (α1<0.85, [10]; α1<0.75, [8]), and reduced scaling exponent α was a strong predictor of mortality [10]. Costa et al indicated a loss of complexity with CHF [15]. A reduction in HR complexity and fractal scaling properties were reported in CHF patients [30]. Alterations in nonlinear HRV have important prognostic implications. Reduced DFA α1 was a good univariate predictor of mortality in patients with severe left ventricular dysfunction after acute myocardial infarction [8]. The exponent predicted both arrhythmic and non-arrhythmic cardiac death. The reduced chaos in heart failure is likely due to contributors and factors that influence heart rate dynamics to lose partial or full stochastic kinetics of ion channel gating and biochemical processes, thereby making HRV less complex and less fractal. Abnormalities in left ventricular contractility and cardiac autonomic function may both contribute to a decrease in nonlinear dynamic complexity in patients with CHF [17]. The quantification of nonlinear properties of HRV provides important information in risk stratification of CHF. Furthermore, alterations in nonlinear HRV parameters have important predictive implications in arrhythmias. While linear HRV parameters showed no differences prior to VT (in ICD patients), compression entropy before the onset of VT in those HF patients suggest that altered HR dynamics may contribute to VT onset, and nonlinear measures of HRV could have value in improving VT sensing and/or providing a warning to ICD patient of forthcoming shocks [14]. Approximate entropy, a nonlinear index of HR dynamics describing the complexity of the behavior of the RR interval, has provided information on vulnerability to develop atrial fibrillation [13]. DFA fractal scaling exponent has predicted fatal cardiovascular events in various populations [12].

### Mechanisms of chaotic properties in beating variability and its effects on arrhythmogenesis

HRV in healthy subjects has important chaotic properties with complexity and fractal properties in RR intervals. HR regulation is one of the most complex systems. RR intervals are influenced by many factors, such as patterns of autonomic nervous system firing, reduced responsiveness to catecholamine and acetylcholine, all of which change during CHF. At the cellular level, the chaotic properties in beating variability are associated with stochastic channel gating, as well as biochemical and molecular processes. Studies support the notion that the stochastic behavior of ion channels could contribute to chaotic beating [76]. Opening of funny channels (I_f_) and L-type calcium channels (I_Ca, L_) depolarizes cells and increases heart rate, whereas opening of acetylcholine-activated potassium channels (I_KAch_) hyperpolarizes cells and reduces heart rate. Stochastic open-close kinetics of these and other ionic channels contribute the chaotic heart rate. Stochastic Ca^2+^ release from sarcoplasmic reticulum, as well as stochastic RyR gating, are also involved in the modulation of chaotic beating rate [77]. Wilders et al. demonstrate the chaotic fluctuations in beat-to-beat interval of pacemaker cells are due to the stochastic open-close kinetics of the gating of membrane ionic channels [78]. Ionic channel turnover represent a stochastic mechanism contributing to such chaotic variations of cellular characteristics. Furthermore, more chaotic variability probably comes from variations in biochemical and molecular processes involving the concentrations of enzymes, metabolites, and second messengers (such as cAMP protein kinase A, phospholamban phosphorylation, and I_Ca, L_ phosphorylation) [77]. Additional biochemical factors involved in the control and modulation of chaotic beating are melatonin, plasma cortisol, growth hormone, catecholamines, angiotensin, renin, aldosterone etc. These factors associated with stochastic channel gating and biochemical processes make HRV more complex and fractal. In CHF dogs, the function of some of these factors that influence RR intervals are turned off or show decreased function, so CHF dogs show decreased complexity and loss of fractal property, which may relate to pathological properties of channels and factors in CHF dogs.

Chaos in HRV decreases with progression of CHF patients [17] and in patients with a propensity for adverse arrhythmic events [20], [59], [60], [61], [62], [63]. Moreover the degree of chaos decreases immediately prior to the onset of ventricular arrhythmias [61], [63], [65]. However, little is known about the mechanisms by which decreased chaos in HRV is arrhythmogenic in the failing heart. Dvir et al [64] demonstrated low chaotic HRV is a predictor for cardiac arrhythmogenic events and that pacing of ventricular tissue in a stochastic rather than in a deterministic rhythm (to enhance chaos) exerted a protective antiarrhythmic effect due to a consequence of inherent chaotic HRV. Stochastic ventricular pacing reduced spatial action potential duration (APD) heterogeneity, discordant APD alternans and wavebreak initiation [64]. These results suggest that the chaos in HRV provides the heart with a protective mechanism against arrhythmogenesis [64].

### Implications

Morning is a time associated with increased workload and physical activity, concomitant with increased sympathetic activity and nonlinear dynamics. Alterations in these parameters with CHF could contribute to increased cardiovascular risk in the morning. The current study characterized heart rate dynamics, autonomic oscillation, and nonlinear dynamics in CHF dogs when there is increased risk of cardiovascular events during morning. Healthy HR fluctuations exhibit fractal-like self-similarity (on time scales ranging from seconds to hours) and complexity, both of which allow for a broad range of adaptive responses. A reduction in HR fractal properties and complexity, lack of morning enhancement of chaotic activity, loss of time-of-day rhythm in autonomic oscillations and nonlinear dynamics, and blunting of the normal morning transition to high heart rate and sympathetic activity could all contribute to altered regulation of the drug-free arrhythmogenic substrate of this new canine CHF model and its morning surge in ventricular arrhythmias. Development of this large animal arrhythmogenic model of CHF that demonstrates a morning surge in ventricular arrhythmias as well as reduced chaos enable us to further define the underlying mechanisms of VT in the failing heart in ways not possible to achieve in humans (i.e. ability to compare CHF to baseline in same animal, absence of drug effects, and later access to tissue & cells for molecular and physiologic studies).

### Conclusion

In this new arrhythmogenic canine CHF model, a morning surge in ventricular arrhythmias was associated with attenuated morning HR rise, blunted autonomic adaptation, reduced cardiac chaos and complexity of heart rate, as well as aberrant time-of-day-dependent variations. This novel large animal model will provide an opportunity for further mechanistic studies not easily done in humans that can directly translate to novel therapeutic approaches in patients.
